# Anti-bacterial efficacy of alcoholic hand rubs in the Kenyan market, 2015

**DOI:** 10.1186/s13756-017-0174-3

**Published:** 2017-01-25

**Authors:** Missiani Ochwoto, Lucy Muita, Keith Talaam, Cecilia Wanjala, Frank Ogeto, Faith Wachira, Saida Osman, James Kimotho, Linus Ndegwa

**Affiliations:** 1 Production Department, Kenya Medical Research Institute, P. O. Box 54840-00200, Nairobi, Kenya; 2 Centers for Disease Control and Prevention, Nairobi, Kenya

**Keywords:** Hand rubs, Hand sanitizer, Efficacy, Organoleptic, Reduction factor

## Abstract

**Background:**

Hand hygiene is known to be effective in preventing hospital and community-acquired infections. The increasing number of hand sanitizer brands in Kenyan hospitals and consumer outlets is of concern. Thus the main aim of this study was to evaluate the anti-bacterial efficacy and organoleptic properties of these hand sanitizers in Kenya.

**Methods:**

This was an experimental, laboratory-based study of 14 different brands of hand sanitizers (coded HS1-14) available in various retail outlets and hospitals in Kenya. Efficacy was evaluated using standard non-pathogenic *Escherichia coli* (ATCC 25922), *Staphylococcus aureus* (ATCC 25923) and *Pseudomonas aeruginosa* (ATCC 27853) as per the European Standard (EN). The logarithmic reduction factors (RF) were assessed at baseline and after treatment, and log reduction then calculated. Ten and 25 healthy volunteers participated in the efficacy and organoleptic studies respectively.

**Results:**

Four (28.6%) hand sanitizers (HS12, HS9, HS13 and HS14) showed a 5.9 reduction factor on all the three bacteria strains. Seven (50%) hand sanitizers had efficacies of <3 against all the three bacteria strains used. Efficacy on E. Coli was higher compared to the other pathogens. Three hand sanitizers were efficacious on one of the pathogens and not the other. In terms of organoleptic properties, gel-based formulations were rated far higher than the liquid based formulations brands.

**Conclusion:**

Fifty percent (50%) of the selected hand sanitizers in the Kenyan market have efficacy that falls below the World Health Organization (WHO) and DIN EN 1500:2013. Of the 14 hand sanitizers found in the Kenyan market, only four showed efficacies that were comparable to the WHO-formulation. There is a need to evaluate how many of these products with <3 efficacy that have been incorporated into the health system for hand hygiene and the country’s policy on regulations on their usage.

## Background

Globally, the prevalence of hospital associated infections (HAIs) ranges from 4 to 10% in developed countries, and has been reported as being more than 20% in developing countries [17]. Studies by Ndegwa et al. [12] established an overall incidence of respiratory HAIs in three major hospitals in Kenya to be 9.2 per 10,000 patient days, with the highest incidence being in the Intensive Care Units (ICUs).

Hand hygiene is known to be effective in preventing hospital and community-associated infections, and a number of studies have demonstrated the benefits of hand sanitizers in both community and hospital settings [5, 10, 15, 16].

Alcohol-Based Hand Rubs (ABHRs) are the most widely used hand sanitizers [16]. They may contain additional active ingredients such as quaternary ammonium compounds (QAC), povidone-iodine, triclosan or chlorhexidine that mainly serve to contribute to the efficacy of formulations [1, 8, 14]. Alcohols act by denaturing proteins, and are most effective at concentrations of 60–80%. Concentrations higher than 80% alcohol are less potent because proteins are not easily denatured in the absence of water [9]. Alcohols manifest a good in vitro germicidal activity against Gram-positive and Gram-negative vegetative bacteria as well as various strains of fungi. However, they have minimal activity against bacterial spores, protozoan oocytes and some non-enveloped (non-lipophilic) viruses [9]. The reference standard against which ABHRs are compared is 60% isopropanol [4]. In most cases, the efficacy of ethanol and isopropanol are comparable, though ethanol has been found to have better efficacy profile against viruses [6]. Some studies have demonstrated that ethanol gel formulations, unless they have been specially formulated and tested, are less efficacious than ethanol solution formulations [2, 7]. There are a number of hand sanitizers sold to the Kenyan market with labels on their package that claim that the handrub can kill 99.9% of germs. The objective of this study was to evaluate the anti-bacterial efficacy and organoleptic properties of the hand sanitizers available in the Kenyan market, to help set the standards required for hand sanitizers in the country.

## Methods

### Study design

This study was an experimental, laboratory-based study that was carried out at the technology development and production facility of the Kenya Medical Research Institute (KEMRI) in Nairobi, Kenya.

### Sample size

**Fig. 1:**
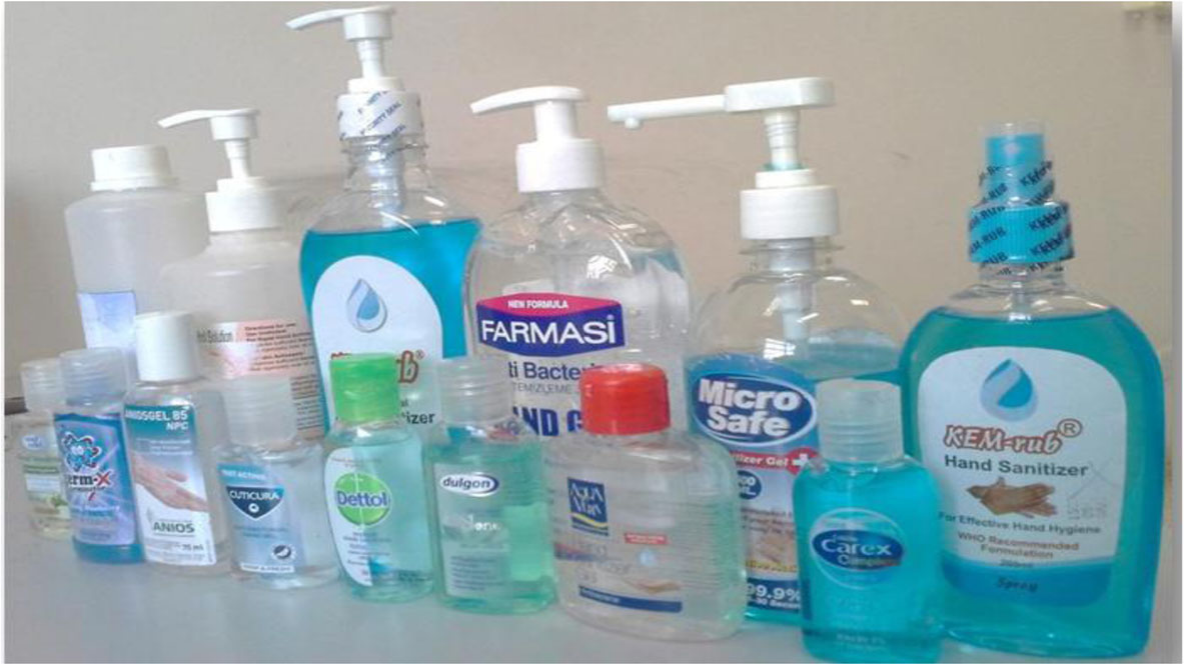
A photograph of hand sanitizers in the Kenyan market that were used in the study

Fourteen (14) available brands of hand sanitizers (Fig. 1) were picked from various retail outlets and hospitals in Kenya. The total number of hand sanitizers, in the market was not available at the time of the study, therefore, the investigators regularly picked up to four different batches of the each hand sanitizer that was in the market from September 2014 to July 2015. Thirty-five healthy volunteers participated in the study: 10 for efficacy and 25 for organoleptic studies.

### Efficacy testing

The number of viable bacterial microbes present after application of the hand rub was used to calculate the efficacy of the hand rub. A hand rub with the ability to reduce the microbes by 50% (equivalent to Log reduction below 3) was considered efficacious. Efficacy testing was carried out step by step as described in the European Standard (EN) 1500:2013; briefly, the standard non-pathogenic *Escherichia coli* (ATCC 25922), *Staphylococcus aureus* (ATCC 25923), and *Pseudomonas aeruginosa* (ATCC 27853) were incubated overnight in a sterile broth suspension.

Ten staff members of KEMRI volunteered to participate in the study and verbal informed consent was sought. The hand rub/sanitizer (HS) samples were fully concealed to the participants; the containers of HS were wrapped with identical opaque papers leaving only the cap of the HS open with codes labelled HS1-14.

All the participants were expected to test all the different batches of HS for all the three pathogens. The initial procedure required the participants to thoroughly wash their hands with soap and water and drying them with paper towels. This was followed by contaminating of 4 fingers in a 10 ml 0.5 Mac Farland suspensions of bacteria (A) prepared as per the method described by the National Committee for Clinical Laboratory Standards [11]. A second set of sterile broth (B) was used to determine the post-value Colony Forming Units (CFU) after sanitizing with respective hand sanitizer. All the hand washing and sanitation were done as described in WHO Hand Hygiene: Why, How & When - brochure of 2009 [18]. Ten microliter (10 μl) of each of suspension was inoculated on Tryptic Soy Agar (TSA) and incubated at 37 °C overnight for pre-value and post-value colony-forming unit (CFU) count respectively. Logarithmic reduction factors (RF) were assessed based on the baseline and after treatment with the HS and the results of each HS were compared with the reference standard (60% IPA). The logarithmic reduction factor was then expressed as a percent reduction. Log reduction was calculated as log_10_ (A) - log_10_ (B) and the percent reduction was calculated as (A-B)/A% where; where A = number of viable microorganism at baseline and B = number of viable microorganism after treatment [4, 18].

### Organoleptic test

A questionnaire was designed to test organoleptic properties of the hand sanitizers in the Kenyan market. The organoleptic properties tested using the questionnaires were: general appearance and feeling of the hand after use and ease-of-use. The 25 selected participants were requested to score the hand sanitizers: 5 as “excellent”, 4 as “good”, 3 as “fair”, 2 as “poor” and 1 as “very poor” and a mean product rating was calculated. During the testing process, the identity of the hand sanitizers was concealed to the participants, by wrapping the containers with opaque papers, leaving only the cap of HS open. This made it difficult for the participants to recognize or speculate the product.

## Results

All hand sanitizer products sampled listed ethanol or isopropyl alcohol as its active ingredient either in single form or in combination with other compounds. We did not do any chemical analysis of the hand sanitizers. Those hand sanitizers that were in single form had different ethanol concentration (70–75%) and they include HS2, HS3, HS4, HS5, HS6, HS7, and HS13 (Table 1). Among ethanol-based hand sanitizers that were in combination with other compounds, there were four different compounds used triclosan (HS1), aloe barbadensis (HS8), chlorhexidine (HS9) and hydrogen peroxide (HS12). Only one hand sanitizer with isopropyl as the active ingredient was in single form (HS14) and the rest were in combination form with triclosan (HS10) and hydrogen peroxide (HS11) (Table 1).

**Table 1:** Log reduction values of various hand Sanitizers in the Kenyan market

Serial No.	Active ingredient	Form	*Escherichia coli* Reduction Factor	*Staphylococcus aureus* Reduction Factor	*Pseudomonas aeruginosa* Reduction Factor
HS 1	Alcohol ^a^and Triclosan	Gel	6.0	3.1	3.8
HS 2	Alcohol^a^	Gel	2.1	1.9	3.0
HS 3	Alcohol^a^	Gel	2.9	1.9	3.0
HS 4	Ethyl Alcohol	Gel	4.8	1.9	3.1
HS 5	Ethyl Alcohol	Gel	3.2	2.1	2.3
HS 6	Alcohol^a^	Gel	3.1	2.3	2.2
HS 7	Alcohol^a^	Gel	3.5	2.9	5.1
HS 8	Ethyl Alcohol and aloe barbadensis	Gel	1.0	0.9	1.5
HS 9	Alcohol and Chlorhexidine	Solution	6.0	5.9	6.1
HS 10	Isopropyl alcohol and Triclosan	Gel	2.3	2.0	2.8
HS 11^b^	Isopropyl alcohol and Hydrogen Peroxide	Gel	1.0	2.1	2.6
HS 12^b^	Ethyl Alcohol and Hydrogen Peroxide	Gel	6.0	5.9	6.1
HS 13	70% Denatured alcohol	Gel	6.0	5.9	6.1
HS 14^b^	75% Isopropyl alcohol	Solution	6.0	5.9	6.1
	70% ethanol	Solution	6.0	5.9	6.1
60% Ref	60% IPA	Solution	3.0	3.0	3.0

^a^Type of alcohol not specified

^b^Locally produced HS

### Active ingredients and effectiveness of the hand sanitizers

Each of the two main active ingredients (ethanol and isopropyl alcohol) had at least one product which demonstrated 99.9% bacterial reduction. Among the ethanol group, only one product with 70% ethanol (HS13) demonstrated a reduction factor of 5.9. The remaining alcohol products (HS2, HS3, HS4, HS5, HS6 and HS7) did not mention the alcohol concentration in the product ingredient list and these products were poorly effective with an overall bacterial reduction factor of less than 3 (Table 1). HS7 was more effective against *Pseudomonas aeruginosa* than the other poorly effective sanitizers whereas HS4 was more effective against *Escherichia coli* than the rest (5.1 and 4.8 bacterial reduction factors respectively) (Table 1).

Among the combined alcohol formulation, two products demonstrated 5.9 overall reduction factor, one combined with chlorhexidine (HS9) and the other combined with hydrogen peroxide (H_2_O_2_) (HS12) (Table 1). On the other hand, one product with alcohol and tricosan (HS1) was effective against *Escherichia coli* (5.9 reduction factor), but was not effective against the other two micro-organisms *Staphylococcus aureus* and *Pseudomonas aeruginosa;* 3.1 and 3.8 respectively. One product with a combination of ethyl alcohol and aloe (HS8) was the least effective among all the products sampled with an overall reduction factor of less than 3. One product (HS14) out of three that contained isopropyl as the only active ingredient had 5.9 reduction factor as compared to those with isopropyl and tricosan (HS10) and isopropyl and hydrogen peroxide (HS11) (Table 1).

Only those hand sanitizers that showed high reduction factor when using the three bacteria strains were considered to be the best and most effective. Based on WHO Requirements for ABHRs (WHO Guidelines on Hand Hygiene in Health Care [17] and (EN) 1500:[4]), seven out of 14 hand sanitizers (50%) had very low efficacy of less than 3 reduction factor, against all the three bacteria strains; *Escherichia coli*, and *Pseudomonas aeruginosa* as compared to *Staphylococcus aureus*.

**Fig. 2:**
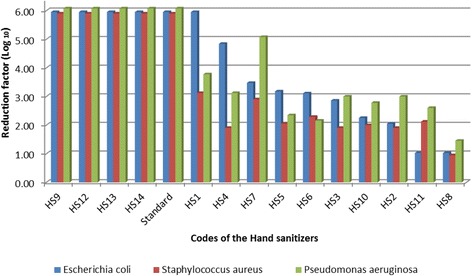
Log reduction of all hand sanitizers against the three bacteria strains

Four hand sanitizers (HS12, HS9, HS13 and HS14) showed 5.8 reduction factor on all three micro-organisms. These results were comparable to those of the World Health Organization (WHO) standard formula. Of these, two were solution formulations and the two were gel formulations, having active ingredients of either alcohol with chlorhexidine or hydrogen peroxide (Fig. 2). A quarter of the hand sanitizers were effective against only one bacteria strain, for example, HS1 and HS4 were so effective on *E. coli*; 5.8 and 4.8 respectively. Whereas HS7 was effective on *P. aeruginosa* (4.8) (Fig. 2). Other sanitizers were not effective in any bacteria, and HS8 was the least effective with *E. coli*; 1.0, *S. aureus*; 0.9 and *P. aeruginosa* 1.5 (Fig. 2). Ethanol-based gel formulations demonstrated higher efficacy profiles than isopropyl alcohol based-gel formulation.

### Organoleptic properties of the hand sanitizers

There were three organoleptic parameters tested in this study; ease-of-use, general appearance and feeling on the hand after use.

**Fig. 3:**
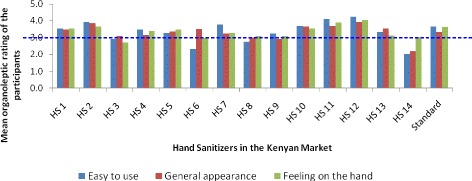
Mean organoleptic comparison of different hand sanitizers in the market

HS11 and HS12 were rated “very good” with a mean of value of 4.1 ± 0.2 and 4.2 ± 0.2 respectively. Most hand sanitizers (9/15) were rated as “good” with a mean range of 3.0–3.9; these were hand sanitizers HS1, HS2, HS4, HS5, HS7, HS9, HS10 and HS13, in the descending order (Fig. 3). Four products were rated as “poor”, with a mean range of 2.0–3.0 and they were hand sanitizer HS3, HS8, HS6 and HS14 (Fig. 3).

Generally, in easy to use, hand sanitizer HS12 and HS11 was rated the highest whereas hand sanitizer HS6 and HS14 was rated the least (Fig. 3).

None of the hand sanitizer had a mean of greater than 4. Almost all hand sanitizers (13/15) were rated between 3.0 and 3.9. HS12 had the highest mean of 3.9 ± 0.2 followed by hand sanitizer HS2 with a mean of 3.8 ± 0.2. In the lower bracket, HS14 and HS9 had the least average rates of 2.19 ± 0.1 and 2.92 ± 0.2 respectively (Fig. 3). There was only one hand sanitizer (HS12) that scored “very good” (4.04 ± 0.2) based on how it felt on the hand after using it.

Comparing all the hand sanitizers and the parameters, it was observed that HS1, HS2, HS10, HS11 and HS12 had average rates that were more than 3.5 of all the three parameters (Fig. 3). Some hand sanitizers had contrasting mean of parameter, with one being poor and the other good. For instance, HS6 in easy to use scored poorly whereas in general appearance it was good. Similarly, HS14 scored very poorly on easy to use, poor on general appearance and good on feeling on the hand after use (Fig. 3).

In general, when the three parameters were averaged and compared, HS12 was rated very good with a score of 4.1 and it was followed by HS11 with a score value of 3.9. The lowest were HS 14, 3, 6 and 8 (Fig. 3).

## Discussions

Use of hand sanitizers has gained popularity in Kenya in the recent past. This has led to the development, production and importation of several hand sanitizers by various companies with the aim of commercialization as well as supporting the health care system in preventing transmission of pathogens.

Four out of 14 (28%) hand sanitizers (HS12, HS9, HS13 and HS14) that were subject of this study showed efficacy profiles that were above the 60% IPA Reference Standard. The four achieve the required high log reduction rate. Seven out of 14 hand sanitizers (50%) had very low efficacy of less than 3.0 against all the three bacteria and hence failing to meet the Health Canadian Requirements for ABHRs of log reduction of ≥3 using EN or ASTM methods [3]. All the poor performing were gel formulations. This finding is in concordance with those of the studies by Kramer et al. [7] and Dharan et al. [2] who established that ethanol gel formulations, unless they have been specially formulated and tested, are less efficacious than ethanol solution formulations. Edmond and Macinga [3] reported a study in Canada that demonstrated that formulation of ABHRs had far greater influence on efficacy than alcohol concentration alone. They established that products having concentration of 70% performed equally well and sometimes better than those with higher concentration. It is this concern that led to the development of solution-based alcohol formulations by WHO for local production in the willing health institutions [17]. However, as demonstrated in this study the solution-based alcohol formulations scored very poorly in terms organoleptic properties in comparison with gel-based alcohol formulations where the gel based brands HS11 and HS12 were rated as ‘very good’ while the WHO-formulation (HS14) was rated as being ‘very poor’. It is notable that the gel-based brands HS12 achieved both high efficacy and desirable organoleptic properties.

The findings in this study that ethanol-based gel formulation (HS12) have higher efficacy than isopropyl alcohol based-gel formulations (HS11), this is contrary to what, has been observed by other studies that isopropyl alcohol solution has higher efficacy than ethanol solution [13].

The main limitation of this study is that we did not pick all known hand sanitizers in Kenya, may be because they were out of stock at the time of the study and the number of hand sanitizers in the market was unknown. To the best of our knowledge we tried as much as possible to sample all hand sanitizers available. Secondly, the EN1500 protocol required that Fingertips of each hand kneaded separately in 10 ml of broth with added neutralizers. We did not add the neutralizers because some HS already contained them. Not knowing the concentration of the active ingredients for many of the products limited our conclusion on the hand sanitizers that poorly performed.

## Conclusion

In conclusion, this study established that 50% of the selected ABHRs in the Kenyan market have the efficacy values below that of International Reference Standard (60% Isopropyl alcohol) and that some of those ABHRs with the desired efficacy value have poor organoleptic characteristics. There is a need to evaluate how many of these products with <50% efficacy that have been incorporated into the health system for hand hygiene and the country’s policy on regulations on their usage.

We recommend that similar experiments to be conducted to involve the other micro-organisms such as viruses and fungi. Additionally, the efficacy studies in relationship to the antimicrobial residual effect may be necessary too.

## Abbreviations

μl: Microliter
ABHRs: Alcohol-based hand rubs
CDC: Centers for Disease Control and Prevention
CFU: Colony forming units
EN: European standard
HAIs: Hospital associated infections
HS: Hand sanitizer
HS1- HS14: Codes of hand sanitizers
ICUs: Intensive care units
IPA: Isopropyl alcohol
KEMRI: Kenya Medical Research Institute
RF: Bacterial logarithmic reduction factors
SSC: Scientific Steering Committee
TSA: Tryptic soy agar
WHO: World Health Organization
